# Identification of serotonin 2A receptor as a novel HCV entry factor by a chemical biology strategy

**DOI:** 10.1007/s13238-018-0521-z

**Published:** 2018-03-14

**Authors:** Lin Cao, Jizheng Chen, Yaxin Wang, Yuting Yang, Jie Qing, Zihe Rao, Xinwen Chen, Zhiyong Lou

**Affiliations:** 1 College of Pharmacy & State Key Laboratory of Medicinal Chemical Biology, Nankai University, Tianjin, 300071 China; 2 National Laboratory of Macromolecules, Institute of Biophysics, Chinese Academy of Sciences, Beijing, 100101 China; 3 State Key Laboratory of Virology, Wuhan Institute of Virology, Chinese Academy of Sciences, Wuhan, 430071 China; 4 School of Medicine and Collaborative Innovation Center of Biotherapy, Tsinghua University, Beijing, 100084 China; 5 Beijing No. 166 High School, Beijing, 100006 China

**Keywords:** HCV, serotonin 2A receptor, entry, antiviral drug

## Abstract

Hepatitis C virus (HCV) is a leading cause of liver disease worldwide. Although several HCV protease/polymerase inhibitors were recently approved by U.S. FDA, the combination of antivirals targeting multiple processes of HCV lifecycle would optimize anti-HCV therapy and against potential drug-resistance. Viral entry is an essential target step for antiviral development, but FDA-approved HCV entry inhibitor remains exclusive. Here we identify serotonin 2A receptor (5-HT_2A_R) is a HCV entry factor amendable to therapeutic intervention by a chemical biology strategy. The silencing of 5-HT_2A_R and clinically available 5-HT_2A_R antagonist suppress cell culture-derived HCV (HCVcc) in different liver cells and primary human hepatocytes at late endocytosis process. The mechanism is related to regulate the correct plasma membrane localization of claudin 1 (CLDN1). Moreover, phenoxybenzamine (PBZ), an FDA-approved 5-HT_2A_R antagonist, inhibits all major HCV genotypes *in vitro* and displays synergy in combination with clinical used anti-HCV drugs. The impact of PBZ on HCV genotype 2a is documented in immune-competent humanized transgenic mice. Our results not only expand the understanding of HCV entry, but also present a promising target for the invention of HCV entry inhibitor.

**Electronic supplementary material:**

The online version of this article (10.1007/s13238-018-0521-z) contains supplementary material, which is available to authorized users.

## INTRODUCTION

Hepatitis C virus (HCV) is a major health threat affecting more than 170 million people worldwide (Webster et al., 2015). Approximately 80% of infections persist to chronicity and lead to liver pathologies, including fibrosis, cirrhosis and hepatocellular carcinoma. Several host factors have been identified to facilitate multistep HCV entry, including CD81 (Pileri et al., 1998), scavenger receptor class B type 1 (SR-BI) (Scarselli et al., 2002), claudin 1 (CLDN1) (Evans et al., 2007), occludin (OCLN) (Ploss et al., 2009), the low-density lipoprotein receptor (LDLR) (Agnello et al., 1999), liver/lymph node-specific intercellular adhesion molecule 3-grabbing integrin (Gardner et al., 2003; Lozach et al., 2003) and glycosaminoglycans (Germi et al., 2002), cholesterol uptake receptor Niemann-Pick C1-like 1 (NPC1L1) (Sainz et al., 2012), receptor tyrosine kinases epidermal growth factor receptor and ephrin receptor A2 (Lupberger et al., 2011), transferrin receptor 1 (TfR-1) (Martin and Uprichard, 2013), serum response factor binding protein 1 (SRFBP1) (Gerold et al., 2015) and very-low-density lipoprotein receptor (VLDLR) (Ujino et al., 2016).

Since HCV was first discovered in 1989, extensive efforts have been launched for anti-HCV therapy intervention. Upon pegylated interferon (PEG-IFN) and ribavirin as the standard therapy for a long time, several direct-acting antivirals (DAAs) have been approved by FDA for anti-HCV therapy in combination with PEG-IFN and ribavirin, leading to improvement of viral clearance rate in patients (Ozaras et al., 2013). However, these DAAs have a low genetic barrier to resistance, side effects, potential for drug–drug interaction, and are costly. Very recently, the risk of hepatitis B virus (HBV) reactivating in some patients treated with DAAs for hepatitis C has been warned (Communication FDS, 2016; De Monte et al., 2016; Wang et al., 2017). Therefore, there remains a great need to improve HCV therapy, analogous to HIV therapy, with combination of antivirals targeting multiple aspects of HCV lifecycle. HCV entry is an essential target step for antiviral development, but FDA-approved HCV entry inhibitor remains exclusive. Most interestingly, from a chemical biology aspect, new antiviral compounds targeting host proteins can be used as chemical probes to provide new vision for uncovering host factors in HCV lifecycle, furthering the understanding of HCV biology.

## RESULTS

### The antagonist of 5-HT_2A_ receptor inhibits HCV proliferation

**Figure 1:**
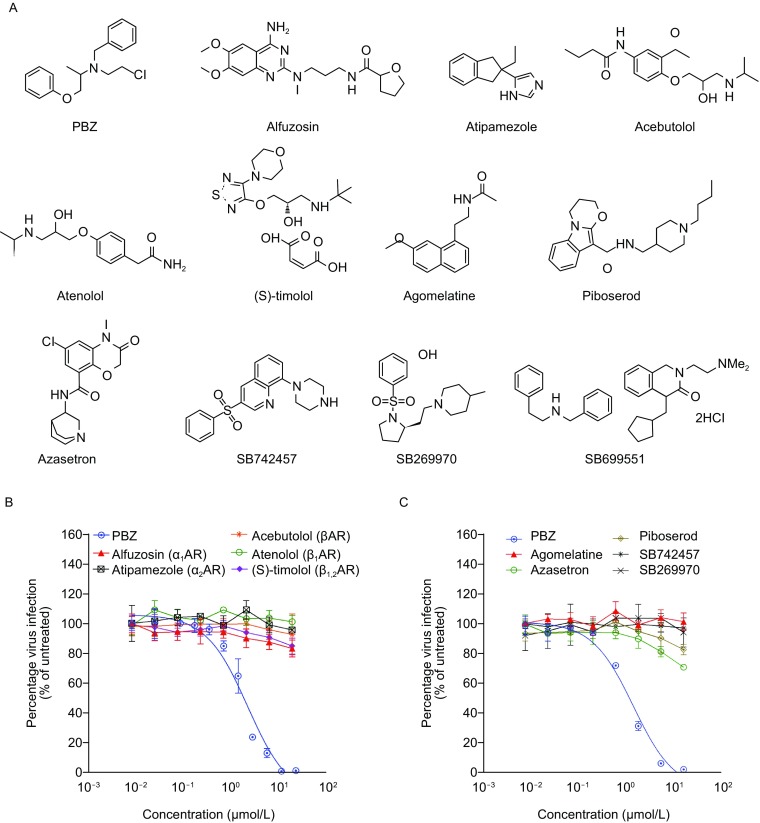
Chemical probes help to identify the antagonism of 5-HT_2A_R inhibiting HCVcc. (A) Chemical probes are composed by a subset of selected FDA-approved adrenergic receptors and serotonin receptors antagonists. (B and C) The HCV inhibition of all chemical probes. Huh7.5.1 cells infected by HCVcc were treated with various adrenergic receptors antagonists (B) and serotonin receptors antagonists (C) at the indicated concentrations at 37 °C for 48 h. The infections are quantified by qRT-PCR measuring virus RNA. All results are graphed as the mean ± SD for triplicate samples

By screening a library composed by FDA-approved drugs, we found that phenoxybenzamine (PBZ) potently inhibits HCV proliferation (EC_50_ value of 1.45 μmol/L) (Fig. 1A and 1B). PBZ is an irreversible antagonist of adrenergic receptors (ARs) and serotonin (5-HT) 2A receptor (5-HT_2A_R), which are two kinds of G protein-coupled receptors (GPCRs) with essential physiological functions (Frang et al., 2001; Doggrell, 1995). This finding prompted our great interest to investigate the association of these two kinds of GPCRs with HCV infection, as well as evaluate their potential for anti-HCV therapy.

Adrenergic receptor family has five major members, α1, α2, β1, β2 and β3 receptors (Bang and Choi, 2015). And the 7 general 5-HT receptor classes include a total of 14 known 5-HT receptors, including 5-HT_1A_, 5-HT_1B_, 5-HT_1D_, 5-HT_1E_, 5-HT_1F_, 5-HT_2A_, 5-HT_2B_, 5-HT_2C_, 5-HT_3_, 5-HT_4_, 5-HT_5A_, 5-HT_5B_, 5-HT_6_ and 5-HT_7_ receptors (Barnes and Sharp, 1999). We first selected other selective antagonists to assess which GPCRs antagonism of PBZ is responsible for HCV inhibition. Alfuzosin (a selective α_1_AR antagonist) (Wilde et al., 1993), atipamezole (a selective α_2_AR antagonist) (Vacher et al., 2010), and acebutolol (a βAR antagonist) (van den Meiracker et al., 1988), atenolol (a selective β_1_AR antagonist) (Baldwin et al., 1986) and (S)-timolol maleate (a β_1_AR and β_2_AR antagonist) (Weinstock et al., 1976) present no inhibition on HCV proliferation, suggesting HCV inhibition of PBZ is not correlated to its antagonism on adrenergic receptors (Figs. 1A, 1B and S1A). Similarly, agomelatine (a 5-HT_2B_ and 5-HT_2C_ receptors antagonist) (Popoli, 2009), azasetron (a selective 5-HT_3_ receptor antagonists) (Tsukagoshi, 1999), piboserod (a selective 5-HT_4_ receptor antagonist) (Brattelid et al., 2004), SB742457 (a selective 5-HT_6_ receptor antagonist) (Upton et al., 2008) and SB269970 (a selective 5-HT_7_ receptor antagonist) (Mahe et al., 2004) do not display HCV inhibition till 20 μmol/L (Figs. 1A, 1C and S1B). SB699551, which is a selective 5-HT_5A_ receptor antagonist (Corbett et al., 2005), also did not present anti-HCV activity at the concentration with no cytotoxicity (Fig. S1C). Together hints the antagonism of 5-HT_2A_R by PBZ is responsible for HCV inhibition.

### 5-HT_2A_ receptor functions in HCV life cycle

**Figure 2:**
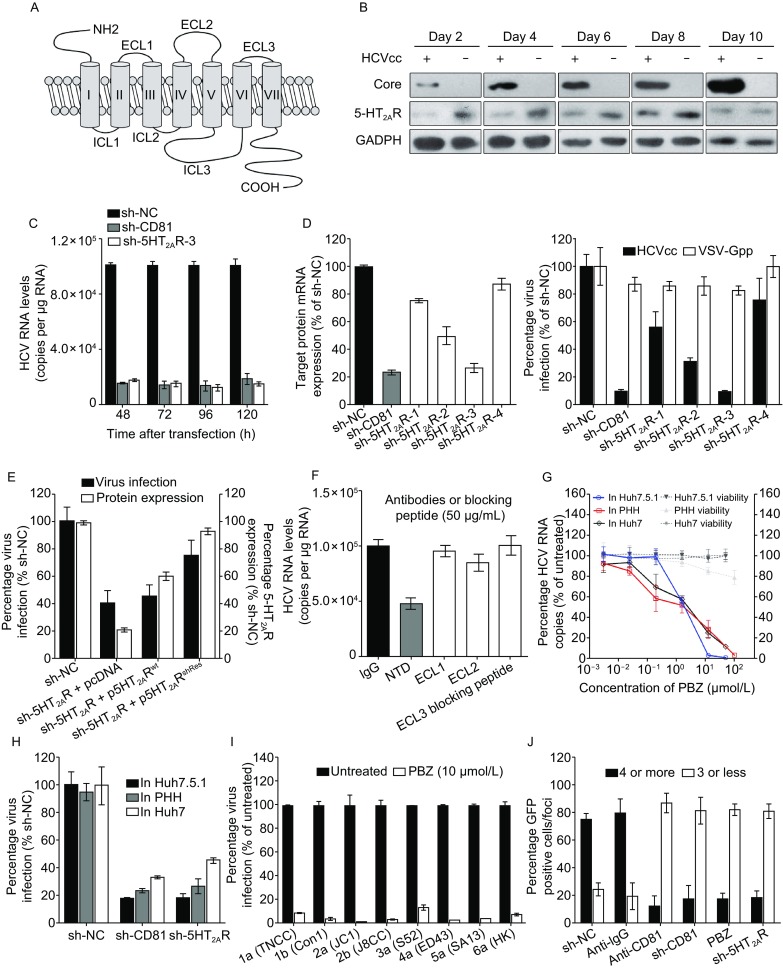
5-HT_2A_R plays a role in HCV infection. (A) The topology of 5-HT_2A_R. (B) Expression of 5-HT_2A_R, HCV core and GADPH in HCV-infected or mock Huh7.5.1 cells. HCVcc at 2 MOI was used to infected cells over the course of 10 days. The expression of host GADPH is shown as a control protein. (C) Silencing of 5-HT_2A_R and CD81 impairs HCV infection. Huh7.5.1 cells were silenced with sh-NC, sh-5HT_2A_R-3 or sh-CD81, followed by HCVcc infection over the course of 5 days. The transcript levels of 5-HT_2A_R and CD81 were quantified by qRT-PCR, normalized to GAPDH and graphed as a percentage of the maximum number of copies determined in sh-NC-containing cells. All results are graphed as the mean ± SD for triplicate samples. (D) The infection of HCVcc, but not VSV-Gpp, is correlated with 5-HT_2A_R expression. Huh7.5.1 cells containing shRNAs or sh-NC were infected with HCVcc or VSV-Gpp at 37 °C for 48 h. The transcript levels were quantified by qRT-PCR, normalized to GAPDH and graphed as a percentage of copies determined in sh-NC-containing cells. The infections were quantified by measuring the luciferase activity in relative luminescence units. Virus infection is expressed as a percentage relative to that in sh-NC-containing cells. (E) HCVcc infection is rescued by 5-HT_2A_R overexpression. Huh7.5.1 cells containing sh-NC, sh-5HT_2A_R and pcDNA empty plasmid, sh-5HT_2A_R and p5HT_2A_R^wt^, or sh-5HT_2A_R and p5HT_2A_R^shRes^ were infected by HCVcc at 37 °C for 48 h. The expression levels of 5-HT_2A_R were examined by Western blot and normalized to GADPH. Virus infection and protein expression are expressed as a percentage relative to sh-NC-containing cells. (F) Huh7.5.1 cells infected by HCVcc in the presence of antibodies or blocking peptide at 50 μg/mL at 37 °C for 48 h. Virus infection is expressed as percentages relative to buffer-treated control cells. (G) PBZ inhibits HCVcc in Huh7.5.1, Huh7 and PHHs. All cells infected by HCVcc were treated with PBZ at the indicated concentrations at 37 °C for 48 h. HCV RNAs are quantified by qRT-PCR and expressed as percentages relative to 0.5% DMSO-treated control cells. (H) Silencing of 5-HT_2A_R in Huh7.5.1, Huh7 and PHHs impair HCV infection. Huh7.5.1 cells, Huh7 cells and PHHs were silenced with sh-NC, sh-CD81 or sh-5HT_2A_R, followed by HCVcc infection at 37 °C for 48 h. HCV RNAs are quantified by qRT-PCR and expressed as percentages relative to sh-NC-containing control cells. (I) Intracellular HCV genome levels detected in Huh7.5.1 cells, which are infected by virus containing the structural region of the indicated genotypes, treated with 10 μmol/L PBZ. The infections are quantified by measuring HCV RNAs for detection by qRT-PCR and expressed as percentages relative to 0.5% DMSO-treated cells. (J) 5-HT_2A_R is required for HCV cell-to-cell spread. Cells containing sh-NC, sh-5HT_2A_R and sh-CD81 were infected with HCV (JFH-1) with an EGFP reporter. At 24 hpi, the cells were washed and incubated in fresh medium containing 1% methyl cellulose. To examine the effect of anti-CD81 mAb to HCV cell-to-cell spread, Huh7.5.1 cells were infected by HCV (JFH-1) with an EGFP reporter. At 24 hpi, the cells were washed and incubated in fresh medium containing anti-IgG or anti-CD81 mAb together with 1% methyl cellulose. At 72 hpi, the number of EGFP-positive cells per foci was counted, and the size of the foci observed is expressed as the average percentage of total foci. All results are graphed as the mean ± SD for triplicate samples. The data presented are representative of three independent experiments

5-HT_2A_R is mainly located in the cortex, claustrum and basal ganglia, but also widely distributes in peripheral and central tissues (Hoyer et al., 2002). It is composed by seven trans-plasma membrane helices with an extracellular N-terminal fragment (NTD), three extracellular loops (ECLs), ECL1, ECL2 and ECL3, and intracellular compositions (Fig. 2A). We first demonstrated that 5-HT_2A_R protein is expressed in Huh7.5.1 cells and primary human hepatocytes (PHHs) (Fig. S2A and S2B). The transcription level of 5-HT_2A_R is comparable to NPC1L1 and LDLR (ranging from 1%–3% of GADPH mRNA), but is markedly less than other HCV receptors or entry factors (Fig. S2B). And without unexception, 5-HT_2A_R is distributed on plasma membrane of liver cells (Fig. S2C), like it is in other cells (Raote et al., 2013).

Similar to what has been observed for other HCV receptors or entry factors (Sainz et al., 2012), we observed down-regulation of 5-HT_2A_R in HCVcc-infected Huh7.5.1 cells. The expression of 5-HT_2A_R protein were remarkably down-regulated within 6 days of HCV infection, but were moderately regained from 8 day infection (Fig. 2B). Because of the correlation between 5-HT_2A_R expression and HCV infection, we next assessed whether 5-HT_2A_R expression affects HCV infection by transfecting Huh7.5.1 cells with shRNAs targeting 5-HT_2A_R or CD81. Compared to cells transfected with an irrelevant shRNA (sh-NC), CD81- and 5-HT_2A_R-silenced cells were significantly less susceptible to HCVcc up to 120 h post-transfection (Fig. 2C). Inhibition of HCV also correlates with a reduction in 5-HT_2A_R expression (Fig. 2D). As sh-5HT_2A_R-3 displays the best silencing and inhibition efficiency, we used this shRNA in the following experiment and referred it as sh-5HT_2A_R hereafter. Moreover, the inhibition is 5-HT_2A_R specific, since the silencing of 5-HT_2A_R does not result off-target effects on the transcriptions of other known HCV receptors or entry factors (Fig. S3). Furthermore, the inhibition is HCV specific, as the treatment of PBZ and the silencing of 5-HT_2A_R had no effect on vesicular stomatitis virus G protein pseudo-typed virus (VSV-Gpp) (Figs. 2D and S4A). Lastly, the effects of the silencing of endogenous 5-HT_2A_R on HCVcc infection are rescued by the RNAi-resistant ectopic expression of wild-type 5-HT_2A_R (Fig. 2E). And the overexpression of 5-HT_2A_R enhances HCV infection (Fig. S5).

We next assessed whether HCV infection was sensitive to inhibition by antibody-mediated blocking of cell surface 5-HT_2A_R. Huh7.5.1 cells were treated with antibodies against 5-HT_2A_R NTD, ECL1 and ECL2 before HCV infection. Because the antibody against ECL3 is not available, we used its blocking peptide to test the impact on HCV infection. We observed that the reagent targeting NTD, but not ECL1, ECL2 or ECL3, suppressed HCV infection (Fig. 2F). Conceivably, the inhibition of antibody was HCV specific, as it had no effect on VSV-Gpp proliferation (Fig. S4B).

To assess whether the function of 5-HT_2A_R in HCV lifecycle is a general observation but is not cell-specific, we further determined the HCV inhibition of PBZ and the silencing of 5-HT_2A_R in Huh7 cells and PHHs. Similar to those have been observed in Huh7.5.1 cells, PBZ treatment and the down-regulation of 5-HT_2A_R inhibited HCV in Huh7 cells and PHHs (Fig. 2G and 2H). Moreover, we observed PBZ sensitivity across a panel of JFH-1 based inter-genotypic HCV clones containing the structural regions of diverse HCV genotypes (1–6) (Fig. 2I).

We next assessed whether 5-HT_2A_R is required for HCV cell-to-cell spread following a reported foci spread assay (Martin and Uprichard, 2013). A focus containing 4 or more GFP-positive cells is taken as evidence of cell-to-cell spread, as a focus containing fewer than 4 cells could result from cell division during the assay (Martin and Uprichard, 2013). The results showed that approximate 80% of the HCV foci contain 4 or more GFP-positive cells in sh-NC-containing cells or the cells treated by anti-IgG mAb, while less than 10% of the foci contain 4 or more GFP-positive cells in the CD81-silenced cells or in the cells treated with anti-CD81 mAb (Fig. 2J). In the 5-HT_2A_R-silenced cells or the cells treated by PBZ, over 80% of the HCV foci contain less than 4 GFP-positive cells, supporting that 5-HT_2A_R function affects HCV cell-to-cell spread (Fig. 2J).

### 5-HT_2A_ receptor facilitates HCV late endocytosis event

**Figure 3:**
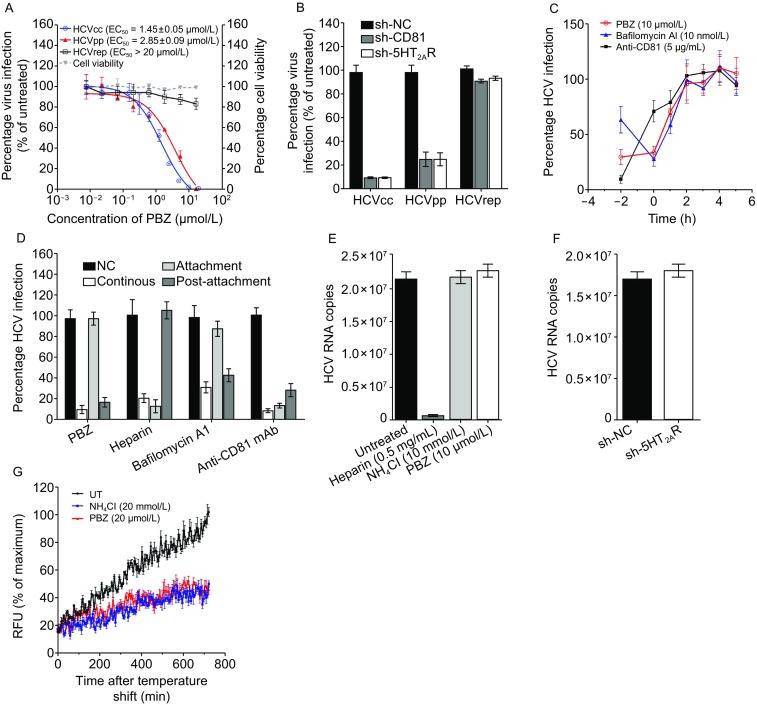
5-HT_2A_R functions in HCV late endocytosis at or before membrane fusion. (A) Inhibitory activities of PBZ on HCVcc (blue line), HCVpp (red line) and HCVrep (black line). Cells infected by HCVcc, HCVpp or that contains HCVrep were treated with PBZ at the indicated concentrations at 37 °C for 48 h. Virus infection and cell viability are expressed as percentages relative to 0.5% DMSO-treated control cells. (B) Cells containing sh-NC, sh-CD81 or sh-5HT_2A_R were infected by HCVcc, HCVpp or transfected with HCVrep and incubated at 37 °C for 48 h. Viral infections are quantified by qRT-PCR and expressed as percentages relative to sh-NC-containing cells. (C) The kinetics of HCV inhibition mediated by PBZ or other reagents was determined by time-of-addition assays. Huh7.5.1 cells were incubated with HCVcc at 4 °C for 2 h (T = − 2). At different time points (T = − 2 to T = 5), PBZ (10 μmol/L), bafilomycin A1 (10 nmol/L) and anti-CD81 mAb (5 μg/mL) were individually added to the cells at 37 °C for 2 h. (D) PBZ inhibits the post-attachment events. Huh7.5.1 cells were infected with HCVcc and incubated at 4 °C for 2 h. Unbound virus was removed by two washes with cold media. Fresh medium was subsequently added, and the cells were shifted to 37 °C to allow synchronous infection. PBZ (10 μmol/L), heparin (1 mg/mL), bafilomycin A1 (5 nmol/L) and anti-CD81 mAb (5 μg/mL) were provided in the media either continuously, during the 4 °C incubation only (initial attachment), or during the 37 °C incubation phase only (post-attachment). Virus infection is expressed as a percentage relative to control cells. (E) PBZ treatment does not affect the binding of HCV to host cells. Huh7.5.1 cells were incubated with wild-type HCVcc along with PBZ (10 μmol/L), heparin (0.5 mg/mL), anti-CD81 mAb (5 μg/mL) or NH_4_Cl (10 mmol/L) in culture at 4 °C for 2 h. Unbound virus was removed by two washes with cold media. The cells were then lysed, and viral RNA was extracted for detection by qRT-PCR. (F) The down-regulation of 5-HT_2A_R does not attenuate the binding of HCV to host cells. Huh7.5.1 cells containing sh-NC or sh-5HT_2A_R were incubated with HCVcc at 4 °C for 2 h. Unbound virus was removed by two washes with cold media. The cells were then lysed, and viral RNA was extracted for detection by qRT-PCR. (G) Huh7.5.1 cells are infected by HCVcc^DiD^ with the treatment of NH_4_Cl (20 mmol/L) and PBZ (20 μmol/L). Results are graphed as a percentage of maximum background-corrected relative fluorescence units (RFU) achieved in 0.5% DMSO-treated control cells. All results are graphed as the mean ± SD for triplicate samples

We next investigate the working step of 5-HT_2A_R in HCV lifecycle. We first observed that both HCVcc and HCV pseudotyped virus (HCVpp) are potently inhibited by PBZ treatment, but HCV subgenomic replicon (HCVrep) activity was not affected in the presence of PBZ at high concentration (Fig. 3A). We also confirmed that the HCV inhibition of PBZ is not side-effected by PBZ-mediated cytotoxicity (Figs. S1A, S1B and S6A), changes in cell proliferation (Fig. S6B), or reduced transcription of known HCV receptors or entry factors (Fig. S6C). Moreover, the proliferation of HCVpp is also significantly attenuated, like HCVcc, by the silencing of 5-HT_2A_R, but in contrast, HCVrep is not sensitive to 5-HT_2A_R down-regulation (Fig. 3B). Furthermore, PBZ and the knockdown of 5-HT_2A_R have no effect on full-length infectious HCVcc RNA replication or secretion of *de novo* HCVcc (Fig. S7A–D). These data show that 5-HT_2A_R plays a role in HCV entry.

We next assessed the entry step that 5-HT_2A_R works on through time-of-addition analysis (Fig. 3C). Huh7.5.1 cells were infected with HCVcc at 4 °C for 2 h (T = − 2 h). After removing unbound viruses, cells were incubated at 37 °C (T = 0 h), and reagents were added to the infected cells at different time points. We selected heparin, anti-CD81 antibody and bafilomycin A1 as controls to represent the typical HCV entry inhibitors working on initial attachment, attachment and the early entry process, and late entry events, respectively (Evans et al., 2007; Liu et al., 2012). HCV is mostly sensitive to bafilomycin A1 at 0–2 h after the 37 °C temperature shift and the HCV inhibiting effects of anti-CD81 antibody decreases at the time of the temperature shift, which is consistent with previous reports (Liu et al., 2012). The growth curve of HCV in the presence of PBZ and bafilomycin A1 is similar, indicating that PBZ acts on the late entry process.

We further verified whether PBZ blocks infection at the initial attachment step or a post-attachment event. PBZ, heparin, bafilomycin A1 and anti-CD81 antibody were either supplied together with HCVcc to cells during the 4 °C attachment step only, and then removed prior to shifting the temperature to 37 °C; or provided only after the temperature shift. The results reveal that all of four reagents suppress HCV infection if they are present throughout the course of the experiment (Fig. 3D). Inhibition by heparin is predominant when it is added during the 4 °C attachment step, whereas bafilomycin A1 is most effective during the post-attachment stage. These are consistent with previous reports (Evans et al., 2007). Again, similar to bafilomycin A1, PBZ strongly inhibits viral infection at post-attachment. Finally, by using heparin and NH_4_Cl (an inhibitor of endosomal acidification and HCV membrane fusion (Sainz et al., 2012) as controls, we observed that PBZ, as well as NH_4_Cl, does not block the binding of HCV virions to host cells, which is in a sharp contrast with heparin (Fig. 3E). And as expected, the silencing of 5-HT_2A_R only negligibly affects the attachment of HCV to cells (Fig. 3F).

We next used a reported fluorescence-based HCV fusion assay (Sainz et al., 2012) to dissect whether 5-HT_2A_R acts at or before membrane fusion. HCVcc was labeled with the hydrophobic fluorophore DiD, which can be anchored on the lipid membrane and will be self-quenching at high concentrations. When viral entry occurs, DiD diffuse away from each other, causing dequenching and allowing for the monitor of virus membrane fusion. The result shows that PBZ inhibits HCVcc^DiD^ fusion as a same level compared to NH_4_Cl till 12 h after binding (Fig. 3G). All these results demonstrated that the antagonism of 5-HT_2A_R inhibits HCV cell entry in the late endocytosis stage at or before virus membrane fusion.

### Antagonism of 5-HT_2A_R reorganize the cellular distribution of CLDN1

We are interested to assess the working mechanism of 5-HT_2A_R in HCV lifecycle. We first hypothesized that 5-HT_2A_R may function as a co-receptor, analogous to CCR5/CXCR4 in HIV entry, and attach HCV virion through the interaction with viral envelop protein(s). But no convinced interaction of 5-HT_2A_R with HCV virion or E1/E2 protein can be detected (data not shown).

**Figure 4:**
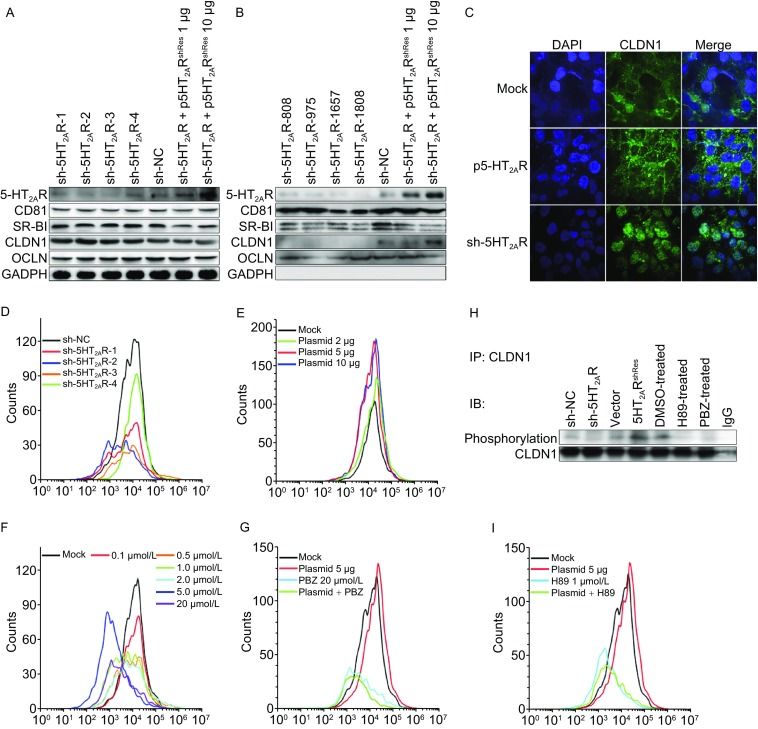
5-HT_2A_R plays a role in the membrane distribution of CLDN1. (A) The expression of 5-HT_2A_R, CD81, SR-BI, CLDN1 and OCLN in Huh7.5.1 whole cells containing sh-NC, sh-5HT_2A_Rs and 5-HT_2A_R overexpression plasmids with indicated amounts. The expression of GAPDH is shown as an internal control. (B) The plasma membranes of Huh7.5.1 cells containing sh-NC, sh-5HT_2A_Rs and 5-HT_2A_R overexpression plasmid were separated and the protein amounts of 5-HT_2A_R, CD81, SR-BI, CLDN1 and OCLN on the plasma membrane were analyzed by Western blot. (C) Cellular distribution of CLDN1 in the 5-HT_2A_R-silenced or overexpressed Huh7.5.1 cells. The localization of endogenous CLDN1 in Huh7.5.1 cells transfected with either an empty vector or 5HT_2A_R, or 5HT_2A_R-silenced cells was observed using confocal microscopy. CLDN1 expressions on the cell membrane in 5-HT_2A_R-silenced cells (D) or 5-HT_2A_R-overexpression cells (E) were assessed by flow cytometry. The situation of CLDN1 positioning on the membrane were plotted, during treatment with PBZ under various concentration (F) or either PBZ (20 μmol/L) (G) or H89 (1 μmol/L) (I) in 5-HT_2A_R-overexpression cells. (H) Exogenous CLDN1 was immunoprecipitated by CLDN1 antibody from indicated cell lysates, and immunoblotted with phosphorylation-Ser/Thr antibodies. The data presented are representative of three independent experiments

We speculated that 5-HT_2A_R may affect other major HCV receptors or entry factors, but the silencing of 5-HT_2A_R and its antagonist PBZ did not negatively regulate the expression and transcription of major HCV entry factors in whole cell (Figs. 4A, S3 and S6C). We thus hypothesized that the loss-of-function of 5-HT_2A_R may alter the correct membrane distribution of HCV receptors or entry factors. We therefore investigated the cellular localization of CD81, SR-BI, CLDN1 and OCLN. With the down-regulation of 5-HT_2A_R, the amount of plasma membrane-located CLDN1 protein has dramatically decreased, while CD81, SR-BI and OCLN have only negligible varieties (Fig. 4B). Moreover, the amount of CLDN1 protein on the plasma membrane is rescued by the RNAi-resistant ectopic overexpression of 5-HT_2A_R (Fig. 4B), like that occurs to HCV infection (Fig. 2E). Flow cytometry further supported that the cell membrane localization of CLDN1 possessed a positive correlation with the expression of 5-HT_2A_R (Fig. 4D and 4E). Furthermore, CLDN1 localizes on the plasma membrane, predominantly at intercellular junctions, whereas CLDN1 was observed in an intracellular location in 5HT_2A_R-silenced cells. As expected, the overexpression of 5-HT_2A_R enhances the membrane localization of CLDN1 (Fig. 4C). Notably, the 5-HT_2A_R antagonism of PBZ puts a brake on the cell membrane localization of CLDN1 in a dose-dependent manner, and suppressed the membrane localization reinforcement of CLDN1 by 5-HT_2A_R overexpression (Fig. 4F and 4G).

A recent work reported that cAMP-dependent protein kinase A (PKA) phosphorylation of CLDN-1 play a role in the cellular distribution of CLDN1 (French et al., 2009), and 5-HT_2A_R is also known to be associated with cAMP/PKA pathway stimulation (Berg et al., 1994; Tamir et al., 1992; Raymond et al., 2001). We next assessed whether 5-HT_2A_R alters the cellular localization of CLDN1 via PKA-dependent phosphorylation of CLDN1. The phosphorylation of CLDN1 in PBZ-treated and 5-HT_2A_R-silenced cells are significantly lower than that in control cells, and is remarkably up-regulated in 5-HT_2A_R-overexpressed cells (Fig. 4H). Moreover, the treatment of H89, an inhibitor of PKA, suppresses the membrane localization reinforcement of CLDN1 by the overexpression of 5-HT_2A_R (Fig. 4I). Notably, the signaling of 5-HT receptors are very complicated and 5-HT_2A_R is also known to regulate many other downstream signaling pathways (Raymond et al., 2001). Whether other 5-HT_2A_R-associated downstream pathway would be involved in HCV lifecycle needs further investigation. Nevertheless, all these results show that the function of 5-HT_2A_R is necessary for the phosphorylation and the correct membrane distribution of CLDN1, thus ensuring HCV natural proliferation.

### Synergistic antiviral effect of PBZ in combination with anti-HCV drugs

**Figure 5:**
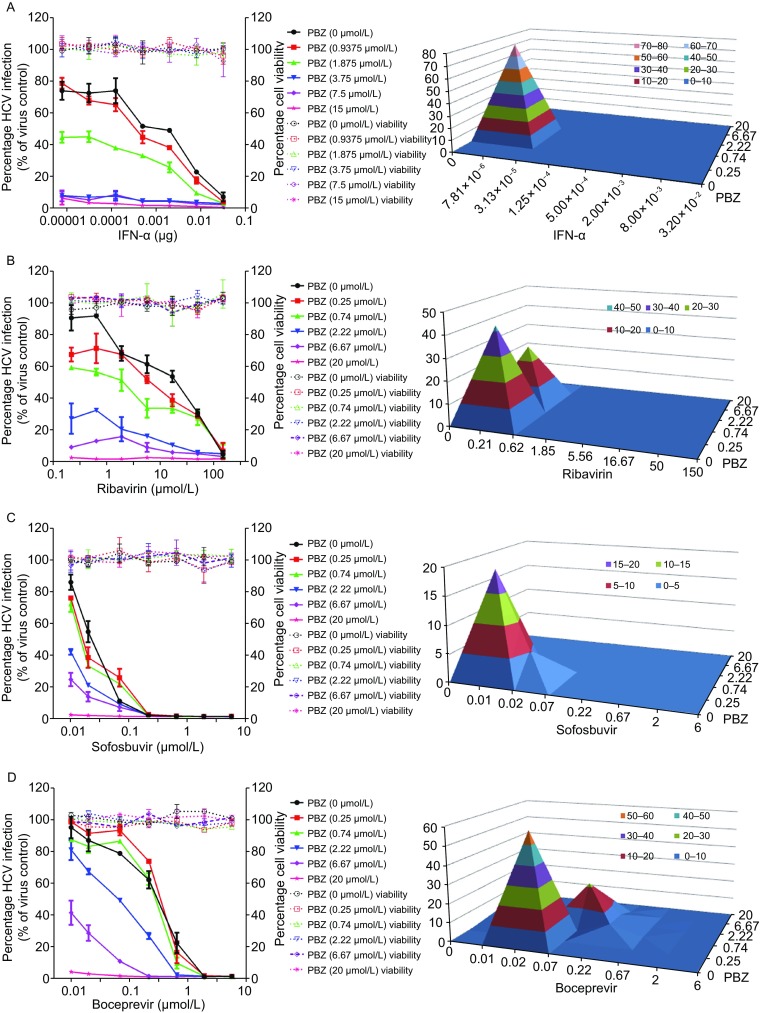

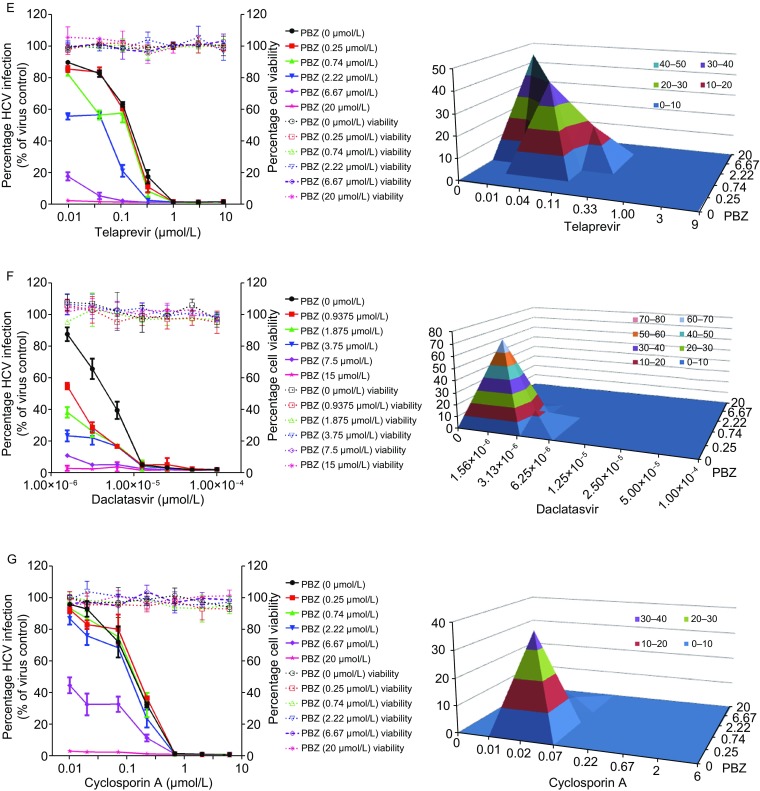
Drug-drug interaction of PBZ with selected different classes of anti-HCV drugs. Huh7.5.1 cells infected by HCVcc were treated with various concentrations of PBZ, IFN-α (A), ribavirin (B), sofosbuvir (C), boceprevir (D), telaprevir (E), daclatasvir (F) and cyclosporin A (G) alone, or in combinations at the indicated concentrations for 48 h. Antiviral activities were determined by measuring the reduction of luciferase activity in the cells. Differential surface plot at the 95% confidence level (CI) were calculated and generated by using MacSynergy II for the drug–drug interaction in the right panels. The 3-dimensional plot represents the differences between the actual experimental effects and the theoretical additive effects at various concentrations of two compounds in combination. Peaks above the theoretical additive plane indicate synergy. Only statistically significant (95% CI) differences between the two compounds were considered at any given concentration. The level of synergy is represented by the log volume values, and color-coded automatically. The level of synergy was defined in MacSynergy as moderate synergy (5 ≤ log volume < 9) and strong synergy (log volume ≥ 9). Results are graphed as the mean ± SD for duplicate samples

Analogous to HIV therapy, we reasoned that the combination of HCV entry inhibitor with different classes of anti-HCV reagents may offer benefits over each single monotherapy. With this purpose, we documented the anti-HCV activity of PBZ in combination with IFN-α, ribavirin, sofosbuvir, boceprevir, telaprevir, daclatasvir and cyclosporin A. We treated HCVcc infected cells with various concentrations of PBZ and other compounds either alone or in combination. The results revealed a concentration-dependent inhibition with all of them alone, or with any two in combination (Fig. 5). The data were further analyzed using a mathematic model, MacSynergy II (Prichard and Shipman, 1990) and revealed that PBZ-IFN-α, PBZ-ribavirin, PBZ-boceprevir, PBZ-telaprevir and PBZ-daclatasvir pairs have strong synergy, while the synergistic antiviral effect of PBZ with sofosbuvir and cyclosporin A is under moderate, according to the suggested criteria. Because the effects on cell viability of all compounds were previously evaluated to ensure that the HCV inhibition was not due to cytotoxicity, it is not surprising that no significant cytotoxicity was observed with the combination of PBZ or either of the other compounds (Fig. 5). All these support the potential usage of PBZ in combination therapy with these drugs.

### PBZ works *in vivo*

**Figure 6:**
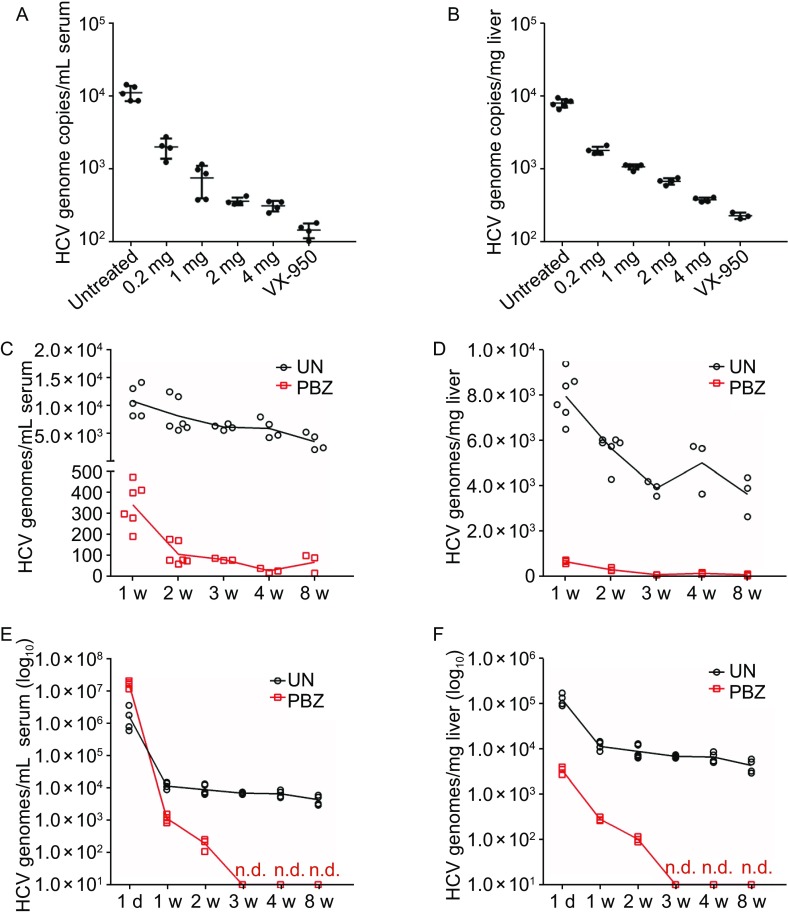
PBZ potently inhibits HCV in transgenic C/O^Tg^ mice. (A and B) HCV RNA levels in serum (genome copies per milliliter of serum) and liver (genome copies per milligram of liver) from mice infected by HCV with subsequent 1 week treatments with various concentrations of PBZ. The treatment of VX-950 with the concentration of 20 mg/kg/d was used as a positive control. (C and D) HCV RNA levels in serum (genome copies per milliliter of serum) and liver (genome copies per milligram of liver) from mice infected by HCV with the treatments of 2 mg/kg/d PBZ for 1 week to 8 weeks. (E and F) HCV RNA levels in serum (genome copies per milliliter of serum) and liver (genome copies per milligram of liver) from mice which PBZ treatment delayed the establishment of HCV infection in mice pretreated for 1 week before infection confirming the ability of this drug to inhibit HCV infection *in vivo*. Samples were analyzed by qRT-PCR. All results are graphed as the mean ± SD for triplicate samples. The data presented are representative of three independent experiments

Finally, to assess the involvement of 5-HT_2A_R in HCV cell entry *in vivo*, we evaluated the ability of ezetimibe to inhibit infection of a genotype 2a (JC-1) in immune-competent humanized transgenic mice of chronic HCV infection harboring both human CD81 and OCLN genes (C/O^Tg^) (Chen et al., 2014). Considering the building up of the persistent infection mouse model in immune-competent mice, we first intravenously inoculated mice with HCV JC-1 for 1 week and subsequently treated mice daily with PBZ at various concentrations. Mice as positive control were treated with VX-950 at 20 mg per kg body weight (mg/kg/d). The treatment of PBZ inhibits HCV infection *in vivo* in a concentration-dependent manner in both serum and liver tissue (Fig. 6A and 6B). Notably, the treatment of 4 mg/kg/d PBZ significantly decreases HCV infection. Moreover, the inhibition of PBZ (2 mg/kg/d) in mice also presents continuously, though HCV genome copies in the infected mice at 2 weeks to 8 weeks are both near the limit of detection (Fig. 6C and 6D). PBZ treatment delayed the establishment of HCV infection in mice pretreated for 1 week before infection confirming the ability of this drug to inhibit HCV infection *in vivo* (Fig. 6E and 6F). Our finding that PBZ can delay the establishment of HCV genotype 2a infection in mice confirms the involvement of 5-HT_2A_R in HCV infection *in vivo* and highlights the therapeutic potential of further pursuing the refinement or development of anti-5-HT_2A_R therapies for the treatment of HCV.

## DISCUSSION

Using a chemical biology strategy, we have demonstrated that 5-HT_2A_R is a HCV entry factor that functions in the late endocytosis stage at or before membrane fusion. To our best knowledge, this is the first time to dissect the role of a GPCR in HCV lifecycle. Notably, CLDN-1 is a HCV entry factor and confers HCVcc and HCVpp entry. CLDN1 does not directly interact with HCV glycoproteins and contributes to the post-binding steps of HCV entry by interacting with CD81 (Evans et al., 2007; Harris et al., 2010; Lindenbach and Rice, 2013). Because 5-HT_2A_R plays a role in the correct plasma membrane localization of CLDN-1 for HCV entry, the impacts to HCVcc and HCVpp, no influence on HCV attachment, and function in HCV late endocytosis of PBZ treatment and 5-HT_2A_R silencing are well fit with reported observations of CLDN1 in HCV lifecycle. Recently, another GPCR antagonist chlorcyclizine (CCZ), a first-generation of H1-antihistamines, was reported to potently inhibit late-stage HCVcc entry, while its action mode is yet clear (He et al., 2015). A significant discrepancy on anti-HCV activity of 5-HT_2A_R antagonist and antihistamines is that PBZ inhibits both HCVcc and HCVpp, but CCZ exhibits no inhibitory effect on HCVpp (He et al., 2015). The dual-inhibition on HCVcc and HCVpp of PBZ is consistent with the crucial role of CLDN1 in HCV entry, and the precise working mode of antihistamines on HCV is warranted further investigation. Lastly, though *in vivo* synergistic anti-HCV activity remains to be determined, the strong synergistic effect of of PBZ in the combination with currently used anti-HCV drugs will probably provide a great potential for the optimal anti-HCV therapy.

## MATERIALS AND METHODS

### Cells

Huh7.5.1 cells were gifted by Apath L.L.C. HEK 293T cells were purchased from the American Type Culture Collection (ATCC) (Manassas, USA). Huh7 cells were gifted by Dr. Wang J. from Peking University. Primary human hepatocytes (PHHs) were purchased from Wuhan Procell Corporation (China). All cells were cultured in Dulbecco’s modified Eagle’s medium (DMEM) (GIBCO, USA) supplemented with 10% (*v*/*v*) fetal bovine serum (FBS) (catalog number 10099-141) (GIBCO, USA), 100 U/mL penicillin, 100 mg/mL streptomycin, and 2 mmol/L l-glutamine at 37 °C in a humidified incubator with 5% CO_2_.

### Primers

The primers used in this study are as following.

**Table:** 

5HT_2A_R-F	5′-CGCGGATCCATGGATATTCTTTGTGAAGAAAATAC-3′
5HT_2A_R-R	5′-CCGCTCGAGTAAATCACACACAGCTCACCTTTTCATTC-3′
sh-5HT_2A_R-1-F	5′-GCCGTAGTGATTATTCTAACTTTCA-3′
sh-5HT_2A_R-1-R	5′-GAGAAGTTAGAATAATCACTACGGCTT-3′
sh-5HT_2A_R-2-F	5′-GGAGGACTATGCAGTCCATCATTCA-3′
sh-5HT_2A_R-2-R	5′-GAGATGATGGACTGCATAGTCCTCCTT-3′
sh-5HT_2A_R-3-F	5′-GGCTCTACAGTAATGACTTTATTCA-3′
sh-5HT_2A_R-3-R	5′-GAGATAAAGTCATTACTGTAGAGCCTT-3′
sh-5HT_2A_R-4-F	5′-GGCCCTGCTCAATGTGTTTGTTTCA-3′
sh-5HT_2A_R-4-R	5′-GAGAGGCCCTGCTCAATGTGTTTGTTT-3′

The primers used for qRT-PCR are as following.

**Table:** 

JFH1-realtime-F	5′-ACTCTATGCCCGGCCATTTGG-3′
JFH1-realtime-R	5′-ACTCGCAAGCGCCCTATCAGG-3′
GAPDH-realtime-F	5′-CCCACTCCTCCACCTTTGACG-3′
GAPDH- realtime-R	5′-CACCACCCTGTTGCTGTAGCCA-3′
CD81-realtime-F	5′-GACCCGCAGACCACCAACC-3′
CD81-realtime-R	5′-CCCAGGAAGCCAACGAACA-3′
CLDN1-realtime-F	5′-CTGTTGGGCTTCATTCTCGC-3′
CLDN1-realtime-R	5′-GGGCGGTCACGATGTTGTC-3′
EGFR-realtime-F	5′-GCCAAGGCACGAGTAACAAG-3′
EGFR-realtime-R	5′-AGGGCAATGAGGACATAACCAG-3′
LDLR-realtime-F	5′-GGACAAATCTGACGAGGAAAAC-3′
LDLR-realtime-R	5′-GCCGCTGTGACACTTGAACTT-3′
NPC1L1-realtime-F	5′-CCAGGGACAAAAGCAAGATG-3′
NPC1L1-realtime-R	5′-CGGGATGACAGATAGCACCA-3′
OCLN-realtime-F	5′-CAAAGGGCTTCATGTTGGC-3′
OCLN-realtime-R	5′-GACTGAGCAGTTGGGTTCACTC-3′
SRBI-realtime-F	5′-CGGGCTGAGCAAGGTTGAC-3′
SRBI-realtime-R	5′-GAAGCGATAGGTGGGGATG-3′
5HT_2A_R-realtime-F	5′-CTATTGCTGGAAACATACTCGTCA-3′
5HT_2A_R-realtime-R	5′-GAGCACGTCCAGGTAAATCCA-3′

### Reagents and antibodies

Alfuzosin hydrochloride (catalog number HY-B0192A), atipamezole hydrochloride (catalog number HY-12380), acebutolol hydrochloride (catalog number HY-17497A), atenolol (catalog number HY-17498), (S)-timolol maleate (catalog number HY-17380), agomelatine (catalog number HY-17038), azasetron hydrochloride (catalog number HY-B0068), piboserod (catalog number HY-15574), SB742457 (catalog number HY-14339), agomelatine (catalog number HY-17038), phenoxybenzamine hydrochloride (catalog number HY-B0431A) and SB269970 (catalog number HY-15370) were purchased from MedChemExpress (USA). SB699551 (catalog number SML0853) was purchased from Sigma (USA). The compounds were initially dissolved in 95% DMSO to a concentration of 10 mmol/L, and the stock solutions were stored at −20 °C. Recombinant human IFNα-2b was purchased from GenScript (China). Heparin sodium was purchased from Rui Taibio (China). Bafilomycin A1 (catalog number IIC2501-0001MG) was purchased from Gene Operation (China). Immediately before addition, these reagents were diluted to the desired concentrations using DMEM (GIBCO, USA) with 10% FBS (GIBCO, USA). The Vybrant^®^ DiD cell-labeling solution (catalog number V22887), which has an excitation wavelength of 644 nm and an emission wavelength of 665 nm, was purchased from Invitrogen (USA). Lipofectamine 2000, lipofectamine 3000 and SuperScript III First-strand Synthesis System for RT-PCR kits were purchased from Invitrogen (USA). The MEGAscript T7 High Yield Transcription kit was purchased from Ambion (USA). The HiScript^®^ II One Step qRT-PCR SYBR^®^ Green Kit (Q221-01) and RNA isolater Total RNA Extraction Reagent (R401-01) were purchased from Vazyme (China). The WST-1 reagent for cell viability assays was purchased from Roche (Swiss). The ATP Determination Kit (catalog number A22066) was purchased from Invitrogen (USA). The ToxiLight™ bioassay kit (catalog number LT07-217) was purchased from LONZA (USA). Fast Mutagenesis System (catalog number FM111-01) was purchased from Transgen Biotech (China).

A monoclonal mouse antibody against the N-terminus of human 5-HT_2A_R (catalog number SC-166775) (Santa Cruz Biotechnology, USA) was used in Western blot detection, immunofluorescence and HCV neutralization. A mouse monoclonal antibody C7-50 against HCV core (catalog number ab2740) was purchased from Abcam (USA). Anti-GAPDH monoclonal antibody (catalog number L018) was purchased from Transgen Biotech (China). Anti-CLDN1 C-Terminal antibody (SAB4503545) for WB and monoclonal antibody (SAB5500083) for flow cytometry and immunofluorescence were purchased from Sigma (USA). The anti-CD81 monoclonal antibody JS81 (catalog number 555675) was purchased from BD Biosciences (USA). The secondary antibodies HRP-conjugated goat anti-mouse IgG (H + L) (catalog number J092) for Western blot analysis were purchased from Transgen Biotech (China), and AlexaFluor 488-labeled goat anti-mouse IgG (H + L) (catalog number A0428) for immunofluorescence were purchased from Beyotime (China). The rabbit polyclonal antibodies to human 5-HT_2A_R ECL1 and ECL2 were obtained from GenScript (China). The rabbit immunogen is peptide-KLH conjugated, and the polyclonal antibodies were purified by sterile filtration affinity purification. The blocking peptide of human 5-HT_2A_R ECL3 (catalog number LS-E26707) was purchased from LifeSpan BioSciences Inc (USA).

### Generation of HCVcc and HCVrep

HCV JFH-1 virus with a luciferase reporter gene (hRluc-JFH1) was constructed according to a previously described method with modifications (Sainz et al., 2012; Qing et al., 2016; Han et al., 2009). Based on the pJFH-1 plasmid gifted by Apath L.L.C., a humanized Renilla luciferase reporter gene was inserted between amino acids 399 and 400 of NS5A in the HCV JFH-1 genome containing four adaptive mutations (core-K78E, E2-G451R, NS3-M260K and NS5A-T462I). The plasmids harboring HCV various subtypes such as 1a (TNCC), 1b (Con1), 2a (JC-1), 2b (J8cc), 3a (S52), 4a (ED43), 5a (SA13) and 6a (HK) were kindly gifts from Pro. Jens Bukh. These plasmids harboring HCV genomes were made through digestion with the *Xba*I restriction enzyme and used as a template for RNA transcription. The transcripts were prepared *in vitro* using the Ambion MEGAscript Kits, and then 10 μg RNA was mixed with 400 μL Huh7.5.1 cells at a concentration of 1 × 10^7^ cells/mL. After electroporation, Huh7.5.1 cells containing viral transcripts were seeded in 10 cm dishes. The cells were cultured and passaged every 3 days until they showed significant apoptosis, and the supernatant was collected and filtered to obtain a stock solution of hRluc-JFH-1 virus. HCV JFH-1 virus with an EGFP reporter gene is an infectious HCV monocistronic reporter virus constructed by inserting an EGFP reporter gene between amino acids 399 and 400 of NS5A of the HCV JFH-1 genome (Han et al. 2009). A stable Huh7.5.1 cell line containing a JFH-1 subgenomic replicon (HCVrep) was created following previously reported methods (Hao et al., 2007; Lohmann et al., 1999). The stable replicon-containing cells were selected and maintained in medium containing 1 mg/mL G418 geneticin (Invitrogen, USA).

### Generation of HCVpp and VSV-Gpp

Pseudotyped viral particles of HCV or VSV were generated by co-transfection of the JFH1 full-length envelope-expressing plasmid (pCDN3.1-JFH1-cE1E2) or the vesicular stomatitis virus glycoprotein (pCDN3.1-VSVG) together with the pNL4-3R-E-luciferase backbone construct into HEK 293T cells as described previously (Cui et al., 2013; Shukla et al., 2010). At 48 h post-transfection, the culture supernatant was harvested, clarified, filtered through 0.45 μm filters (Millipore, USA) and tested for luciferase activity to standardize the viral input in the subsequent inhibition analysis.

### Virus titration

To obtain the titers of viruses containing luciferase reporter genes, the virus stocks were serially diluted 1:10 and incubated with Huh7.5.1 cells for 48 h at 37 °C. The cells were then harvested, and the luminescence was detected according to the manufacturer’s protocol of the Renilla-Glo Luciferase or Bright-Glo Luciferase Assay System. The titers of wild-type HCVcc or that containing an EGFP reporter gene were determined by endpoint dilution assays (EPDA) using focus-forming units (ffu) (Zhong et al., 2006). Huh7.5.1 cells (1 × 10^4^) were seeded per well in 96-well microtiter plates. After an overnight culture, the viruses were serially diluted 10-fold with DMEM containing 10% FBS (10^−1^ to 10^−8^-fold dilutions) and used to infect Huh7.5.1 cells. The plates were then incubated at 37 °C in 5% CO_2_. For viruses containing EGFP reporter genes, the EGFP expression levels were observed using an epifluorescence microscope after 3 to 4 days. For wild-type virus, an antibody against the HCV core protein was used to mark the virus. The determination of the virus titer was shown as the 50% tissue culture infectious dose (TCID_50_). The multiplicity of infection (MOI) is the average number of viruses (TCID_50_) per cell.

### Virus inhibition assay

Cells or cells containing an HCV (JFH-1) subgenomic replicon were seeded in 96-well plates at a density of 1 × 10^4^ cells per well and cultured at 37 °C overnight. The initial concentration of the compounds was 20 μmol/L and serial dilutions were made. For HCVcc, HCVpp or VSV-Gpp, the serially diluted compounds were mixed with a certain titer of virus and added to different cells. For the inhibition of HCVrep, the serially diluted compounds were mixed with the stable Huh7.5.1 cell line containing a subgenomic replicon, followed by 2 days of culture at 37 °C. The amounts of virus were detected by the luminescence or qRT-PCR. EC_50_ is the concentration of the compound at which the luminescence or HCV RNA levels is reduced by 50%. The EC_50_ values were calculated using GraphPad Prism 5.0 software.

### Cell viability assay

Cells were seeded in 96-well plates at a density of 1 × 10^4^ cells per well and cultured at 37 °C overnight. The cells were incubated with the serially diluted compounds or proteins for 48 h. The cell viability was determined in 96-well tissue culture plates using the cell proliferation reagent WST-1 (Roche, Swiss), and the absorbance (OD_450_/OD_630_) was measured to detect the cytotoxicity of compounds according to the manufacturer’s protocol.

### DNA constructs

The human 5-HT_2A_R-pUC57 gene (GenBank: NM_000621.4) was obtained from Genscript (China). The 5-HT_2A_R gene was amplified and cloned with the primers 5HT_2A_R-F and 5HT_2A_R-R as a *BamHI*/*Xhol* fragment into *BamHI*/*Xhol*—digested pcDNA3.1 for overexpression.

### RNA interference and infections

The shRNAs targeting human 5-HT_2A_R (pGPH1/GFP/Neo-HTR2A-Homo-1, pGPH1/GFP/Neo-HTR2A-Homo-2, pGPH1/GFP/Neo-HTR2A-Homo-3, pGPH1/GFP/Neo-HTR2A-Homo-4) were purchased from GenePharma (China). Cells were seeded in 24-well plates at a density of 1 × 10^5^ cells per well. After culture at 37 °C overnight, cells were transfected with sh-5HT_2A_R or sh-NC plasmids by lipofectamin 2000 as per the manufacturer’s instructions. After 48 h, the cells were incubated in selection medium containing 1 mg/mL G418 geneticin. After 24 h, cultures were either mock-inoculated or inoculated with HCVcc at indicated times post-transfection. Total cellular RNA was harvested for extraction in RNA isolater Total RNA Extraction Reagent as the manufacturer’s instructions for qRT-PCR analysis.

### Silencing of endogenous expression of 5-HT_2A_R

The shRNA recombinant lentiviruses targeting human 5-HT_2A_R and CD81 were purchased from Shanghai GenePharma (China), and the virus titers were determined to be 1 × 10^8^ TU/mL. The shRNA recombinant lentiviruses were incubated with 5 μg/mL polybrene to enhance the lentivirus infection and added to PHHs at an MOI of 5. For the knockdown cell lines, PHHs were incubated in selection medium containing 5 μg/mL puromycin (Invitrogen, USA) beginning at 24 hpi. The mRNA levels of the target proteins in knockdown cells were confirmed at 96 hpi by qRT-PCR. The knockdown cell lines were stable after about five days of cell culture and were then used for experiments.

### The production of highly infectious and DiD-labeled HCV virions

The supernatants of wild type HCV (JFH-1) virus cultures were collected, centrifuged to remove cell debris, filtered through 0.45 μm filters (Millipore, USA). Viral supernatants were concentrated by PEG precipitation. Briefly, 40 mL filtered viral supernatants were incubated overnight with 8% (*w*/*v*) PEG followed by centrifugation (8,000 ×*g*, 20 min) and resuspended in 10 mL of spent supernatant. Virus was centrifuged (8,000 ×*g*, 15 min) and resuspended in a final volume of 1 mL of cell culture DMEM. Virus was labeled by adding 5 μL (5 μmol/L final concentration) of lipophilic dye DiD. Virus and dye were incubated for 1 h with shaking at 37 °C while protected from light with shaking. To remove the unbound DiD, the labeled viruses were buffer-exchanged and concentrated to 200 μL in PBS by ultra-filtration with a MW cutoff of 100 kDa three times, with 20 mL PBS added each time. The labeled viruses were collected and subjected to viral titration assays using focus formation assays or RNA measurements by qRT-PCR. The concentrated DiD-labeled HCV was used for subsequent fusion studies.

### HCVcc^DiD^ fusion assay

Huh7.5.1 cells were seeded per well in 48-well microtiter plates. After an overnight culture, cells were treated for approximately 2 h with indicated concentrations of 0.5% DMSO, NH_4_Cl and PBZ at 37 °C. Cells were incubated on ice for 10 min before the addition of DiD-HCV (MOI = 1). For DiD dequenching analysis, vehicle-treated or treated cultures were mock-infected with cDMEM^DiD^ or infected with HCVcc^DiD^ JFH-1 at an MOI of 5 for 1 h at 4 °C. After washing to remove unbound viral particles, plates were brought to 37 °C and immediately placed in a Fluostar OPTIMA microplate reader (Thermofisher, USA) and DiD dequenching was measured every 6 min over the course of 12 h at 37 °C at 640 nm (excitation) and 670 nm (emission) for 120 cycles in kinetic mode.

### Cell-to-cell transmission

HCV cell-to-cell spread was measured according to a previously described method, with modifications (Gondar et al., 2015). Huh 7.5.1 cells were seeded in 24-well plates at a density of 1 × 10^5^ cells per well. After culture at 37 °C overnight, Huh 7.5.1 cells were infected with HCV JFH-1 virus with an EGFP reporter at an MOI of 0.01. For shRNA-containing Huh7.5.1 cells, at 24 hpi, the cells were washed and incubated in fresh medium containing 1% methyl cellulose (Sigma, USA), which prevents cell-free viral spread. For Huh7.5.1 cells, at 24 hpi, antibodies against CD81 or an isotype control antibody were added to the culture medium together with 1% methyl cellulose. At 72 hpi, the cells were observed using an epifluorescence microscope to visualize the expression of EGFP.

### RNA analysis

Total cellular RNA was isolated with TRIzol reagent according to standard protocols. One-step qRT-PCR was performed with HiScript^®^ II One Step qRT-PCR SYBR^®^ Green Kit (Vazyme, China) using an Applied Biosystems 7500 real-time thermocycler (Applied Biosystems, USA). The target gene and GAPDH transcript levels were determined using the ΔΔCT method. To determine the amount of purified JFH-1 virions, the pUC18-JFH1 plasmid was used as a standard sample to generate a standard curve ranging from 10^3^–10^11^ copies/mL. JFH-1 RNA copies were quantified using the HiScript^®^ II One Step qRT-PCR SYBR^®^ Green Kit (Vazyme, China).

### Western blot analysis

Cells were harvested in lysis buffer containing 50 mmol/L Tris-HCl (pH 8.0), 150 mmol/L NaCl, 1% Nonidet P-40, 0.1% SDS, 2 mmol/L EDTA and a protease inhibitor cocktail (Roche, Swiss). The proteins were resolved by SDS-PAGE and transferred to nitrocellulose membranes (Millipore, USA). The membranes were sequentially blocked overnight at 4 °C with 5% nonfat dry milk solution in Tris-buffered saline (TBS). The membranes were then blotted with specific primary antibodies for 1 h and washed 3 times for 5 min with TBS containing 0.1% Tween-20 (*v*/*v*), followed by incubation with secondary antibodies conjugated to horseradish peroxidase for 45 min and washed 5 times for 5 min with TBS containing 0.1% Tween-20 (*v*/*v*) at room temperature. The proteins were visualized by chemiluminescence by using a Clarity Western ECL Substrate (Bio-rad, USA).

### Immunofluorescence microscopy

The cells were fixed with 4% paraformaldehyde for 15 min at room temperature, washed, and permeabilized with 0.05% Triton X-100 in PBS for 10 min. The cells were then washed and blocked with 2% BSA in PBS for 30 min, followed by 1 h incubation with primary antibodies targeting 5-HT_2A_R or CLDN-1. After three washes with PBS, the cells were incubated with secondary antibodies for 1 h. After washing with PBS, nuclei were stained with DAPI. Images were captured using a Nikon A1 Confocal Microscope.

### Binding of virus to host cells

The virus binding assay was performed using a previously reported protocol, with modifications (Qing et al., 2016). Huh7.5.1 cells were seeded at 1 × 10^5^ cells per well overnight. Then the cells were incubated with 0.5% DMSO or 10 μmol/L PBZ for 24 h. The following day, the culture medium was removed and the cells were washed with cold phosphate-buffered saline (PBS). HCV JFH-1 virus was subsequently diluted in 500 μL of DMEM complete medium (dilution fold = 1:2) (2 × 10^6^ HCV RNA copies) and added to the cells. After 2 h of incubation at 4 °C, the unbound virus was removed by three wash steps with 500 μL of PBS, and the cells were lysed in with 500 μL TRIzol. Viral RNA was extracted and detected by qRT-PCR. Huh7.5.1 cells containing sh-NC or sh-5HT_2A_R were seeded at 1 × 10^5^ cells per and virus binding was detected following the above method.

### Animal study

Animal welfare and experimental procedures were carried out in accordance with the Guide for the Care and Use of Laboratory Animals (Ministry of Science and Technology of China), and were approved by the Animal Ethics Committee of Wuhan Institute of Virology. We used 10- to 12-week of age- and gender-matched mice for *in vivo* experiments. Human CD81 and occludin transgenic mice C/O^Tg^ mice (Chen et al., 2014) were divided in 3 batches and injected with HCV (JC-1 strain). A total of four to six mice were included in each group. Because C/O^Tg^ mice are immune-competent, we use a method as infection and treatment by considering the sustainability of the persistent infection mouse model in C/O^Tg^ mice. For infection *in vivo*, mice were tail-vein injected with 1 mL HCV at TCID_50_ = 5 × 10^7^ in 60–120 s to avoid liver injury. For monotherapy, PBZ, VX-950 (Vertex Pharma, MA) or DMSO (Sigma, USA) were intraperitoneal injection administrated daily at one week post HCV infection. On day 0, we intravenously inoculated mice with JC-1. One week later, mice were treated daily with 0.2, 1, 2, or 4 mg PBZ per kg body weight via peritoneal injection for a total of 1–2 weeks. Negative control mice were treated via peritoneal injection with DMSO alone (100 µL per 20 g body weight) and positive control mice were treated with 20 mg per kg body weight VX-950 in DMSO (100 µL per 20 g body weight). Liver tissues and sera were collected at indicated time. The sera (300 μL for each mouse) were collected under anesthesia by extracting the eyeball blood. Mice were then sacrificed for liver tissues for HCV genome measurements. Total RNA was extracted from liver or serum using Trizol or Trizol LS reagent (Invitrogen, USA). qRT-PCR analyses with Artus HCV RG RT-PCR kit (Qiagen, Germany).

### Flow cytometry

Flow cytometry was performed in LSR Fortessa (BD Biosciences, NJ) after cells (1 × 10^6^) were incubated with CLDN1 antibody (10 μg/mL each) 1 h followed by the treatment with FITC-goat anti rabbit second-antibody (10 μg/mL each) for 30 min in PBS containing 2% FBS. Data were analyzed with FlowJo Software (Tree Star, USA) and represented three independent experiments.

### Membrane separation

Cell membranes and cytosolic fractions were freshly prepared from cells by using a Membrane Protein Extraction Kit (Thermofisher, USA). Briefly, cells were resuspended and centrifuged at 300 × *g* for 5 min. The cell pellet was washed with cell wash buffer for twice. After carefully removed the supernatant, 0.75 mL of permeabilization buffer was added to the cell pellet, vortex shortly, and then incubates 10 min at 4 °C with constant mixing. Centrifuge permeabilized cells for 15 min at 16,000×*g*. The supernatant containing cytosolic proteins was carefully removed and transferred to a new tube. 0.5 mL of solubilization buffer was added to the pellet, and resuspended by pipetting up and down and incubate tubes at 4 °C for 30 min. Centrifuge tubes at 16,000×*g* for 15 min at 4 °C. Transfer supernatant containing solubilized membrane and membrane-associated proteins to a new tube for further analysis.

### Interaction of PBZ with other inhibitors of HCV proliferation

Huh 7.5.1 cells were seeded at 3 × 10^4^ cells per well in 96-well microtiter plates. After an overnight culture at 37 °C, a different final concentration of PBZ (0, 0.25, 0.74, 2.22, 6.67, and 20 μmol/L) was added to each row of the 96-well plate. Simultaneously, a different final concentration of other HCV inhibitors was added to each column of the plate. Huh 7.5.1 cells were infected with the HCVcc virus after treatment with the two inhibitors and incubated for 48 h. Antiviral activities were determined by measuring the reduction of luciferase activity in the cells.

## Electronic supplementary material

Below is the link to the electronic supplementary material.

Supplementary material 1 (PDF 795 kb)
