# Supplementary material for: Identification of serotonin 2A receptor as a novel HCV entry factor by a chemical biology strategy
Source: Protein Cell. 2018 Mar 14;10(3):178–95. doi: 10.1007/s13238-018-0521-z (PMC6338621; doi:10.1007/s13238-018-0521-z)
Supplement: Supplementary file 1 — Supplementary material 1 (PDF 795 kb) [file 13238_2018_521_MOESM1_ESM.pdf]

Supporting Information for

Identification of serotonin 2A receptor as a novel HCV entry factor by a chemical biology strategy

Supplementary Figures

S1 Fig

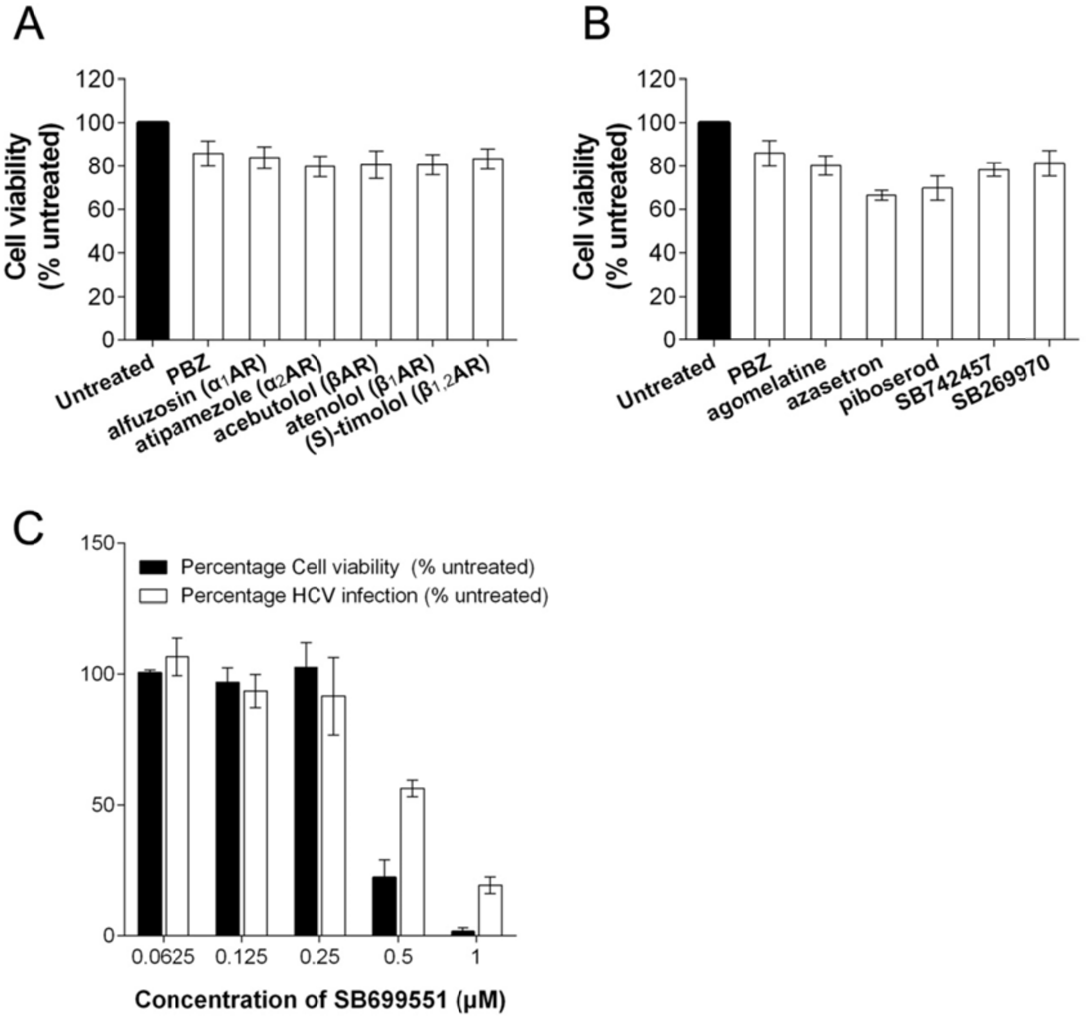

S1 Fig. Cell variability of all chemicals and HCV inhibition of SB699551. (A, B) Huh7.5.1 cell variability in the presence of all chemical probes. Huh7.5.1 cells were treated with adrenergic receptors antagonists **(B)** and serotonin receptors antagonists **(C)** at the concentration of 10 μM at 37 °C for 48 h. Cell viability are expressed as percentages relative to 0.5% DMSO-treated control cells. All results are graphed as the means ± s.d. for triplicate samples. The data presented are representative of three independent experiments.

S2 Fig

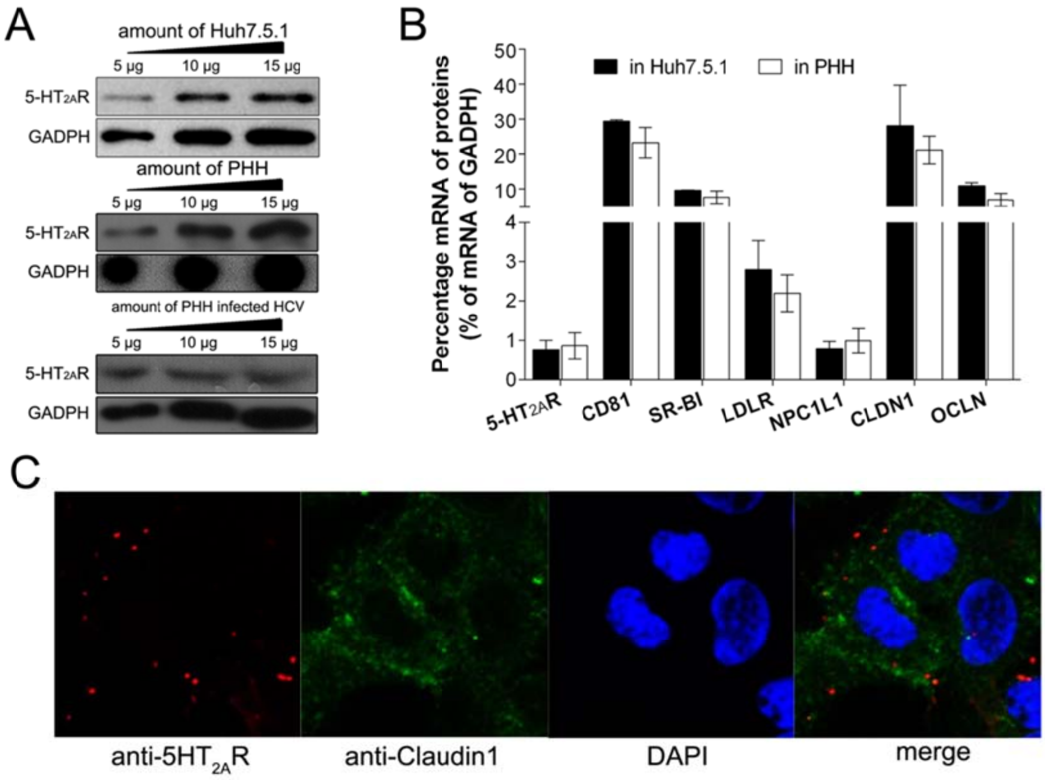

S2 Fig. 5-HT$_{2A}$R is expressed in Huh7.5.1 and PHH. (A) Protein expression of 5-HT$_{2A}$R in Huh7.5.1 cells, PHHs and PHHs are infected HCV virus. Indicated amounts of cells were analyzed by SDS-PAGE. The expression of host GADPH is shown as an internal control. **(B)** The transcription levels of 5-HT$_{2A}$R and known HCV receptors/entry factors in Huh7.5.1 cells and PHHs. The mRNA copies of 5-HT$_{2A}$R, CD81, SR-BI, LDLR, NPC1L1, CLDN1 and OCLN were evaluated by qRT-PCR. The data are expressed as a percentage relative to mRNA copies of host GADPH. All results are graphed as the means ± s.d. for triplicate samples. **(C)** Cellular distribution of 5-HT$_{2A}$R and Claudin-1. Huh7.5.1 cells were fixed, stained with primary anti-5-HT$_{2A}$R antibody and Cy3-labeled secondary antibody (red), and analyzed by confocal microscopy. Claudin-1 were stained by green. Nuclei were stained by DAPI (blue).

S3 Fig

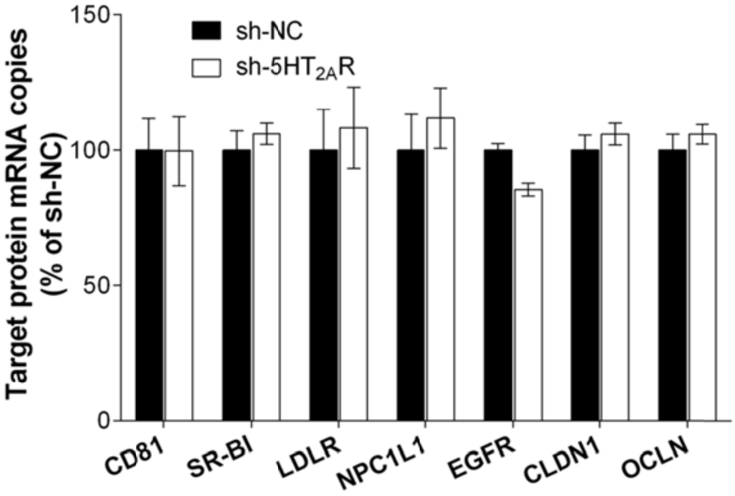

S3 Fig. Silencing of 5-HT$_{2A}$R does not down-regulate the transcription of other reported HCV receptors/entry factors. Huh7.5.1 cells were silenced with sh-NC or sh-5HT$_{2A}$R. The mRNA copies of CD81, SR-BI, LDLR, NPC1L1, CLDN1, EGFR and OCLN were evaluated by qRT-PCR. The data are expressed as a percentage relative to sh-NC transfected cells. All results are graphed as the means ± s.d. for triplicate samples. The data presented are representative of three independent experiments.

S4 Fig

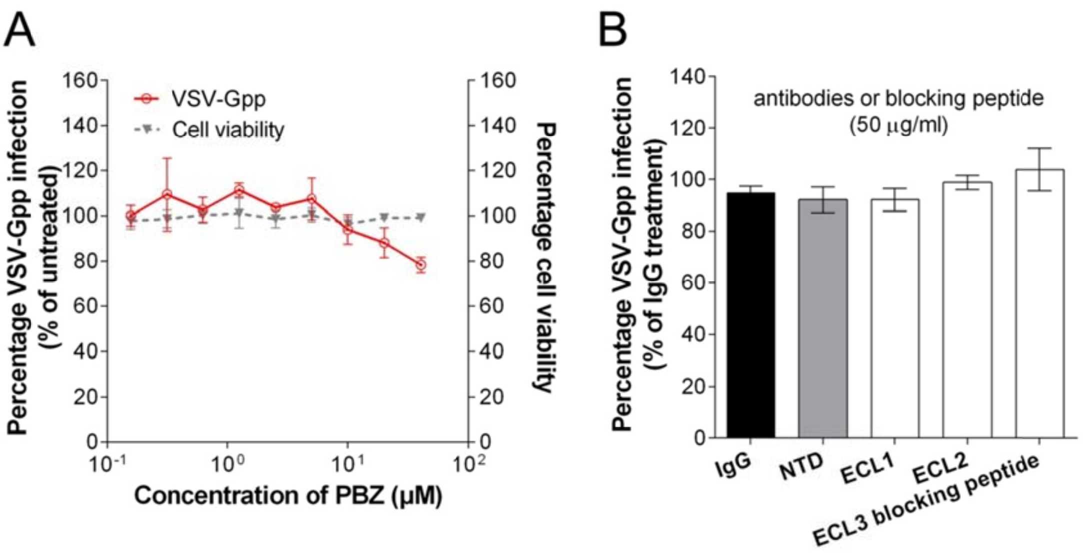

S4 Fig. PBZ, mAb or blocking peptide does not inhibit VSV-Gpp. Huh7.5.1 cells were infected by VSV-Gpp in the presence of PBZ **(A)**, antibodies or blocking peptide **(B)** at the indicated concentrations at 37 °C for 48 h. Virus infection and cell viability are expressed as a percentage relative to DMSO- or buffer-treated control cells. All results are graphed as the means ± s.d. for triplicate samples. The data presented are representative of three independent experiments.

S5 Fig

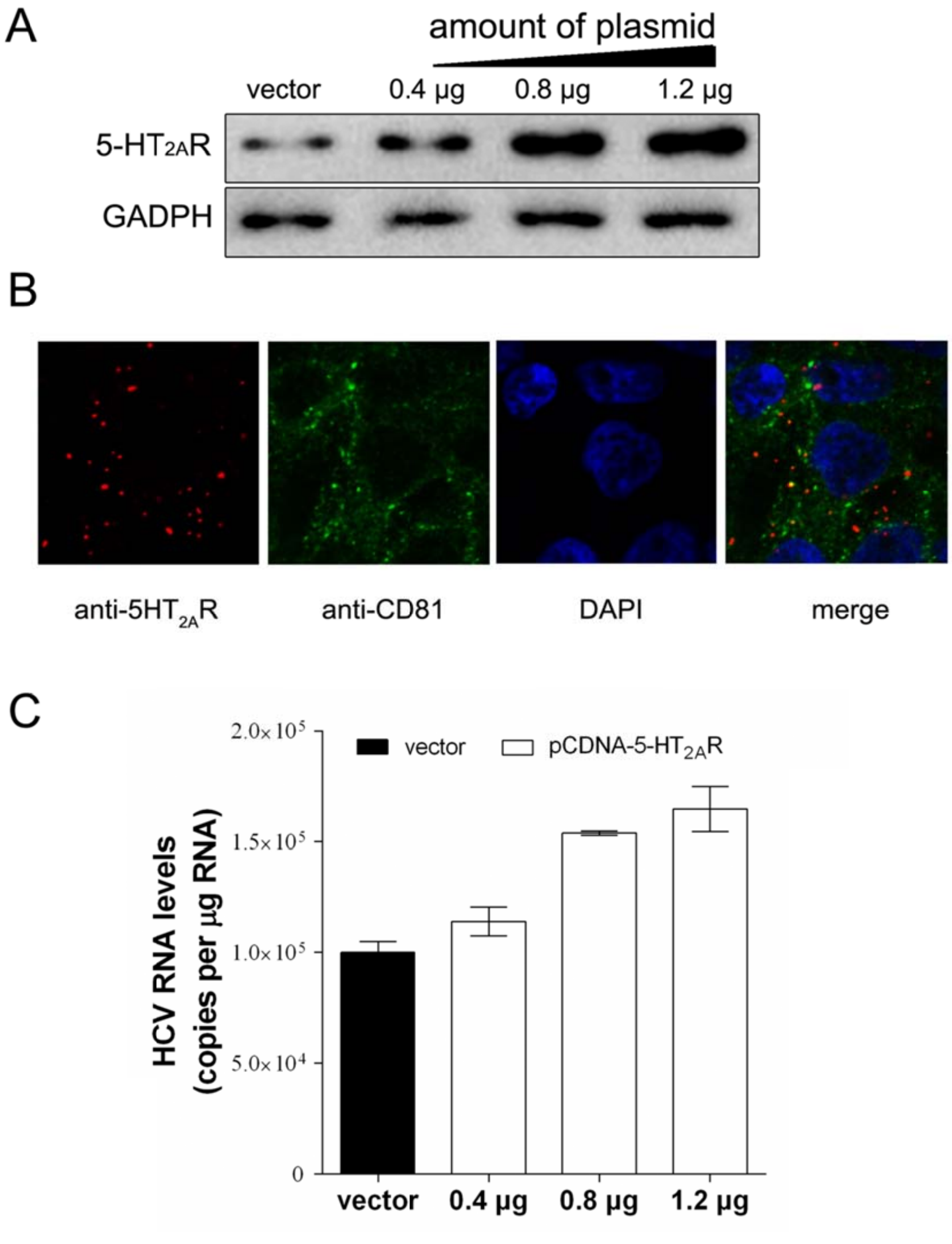

S5 Fig. Overexpression of 5-HT$_{2A}$R enhances HCV infection. (A) Overexpression of 5-HT$_{2A}$R in Huh7.5.1 cells. Indicated amounts of 5-HT$_{2A}$R^{wt} overexpression plasmid (p5HT$_{2A}$R^{wt}) cells

were transfected into Huh7.5.1 cells and the expression of 5-$HT_{2A}R$ were analyzed by SDS-PAGE. The expression of host GADPH is shown as an internal control. **(B)** Cellular distribution of overexpressed 5-$HT_{2A}R$ protein. Plasmid-transfected Huh7.5.1 cells were fixed, stained with primary anti-5-$HT_{2A}R$ antibody and Cy3-labeled secondary antibody (red), and analyzed by confocal microscopy. Claudin-1 were stained by green. Nuclei were stained by DAPI (blue). **(C)** Huh7.5.1 cells were transfected with $p5HT_{2A}R^{wt}$ at the indicated amounts, followed by infection with HCVcc at 37 °C for 48 h. HCV infections were quantified by measuring the qRT-PCR and normalized to GAPDH. Virus infection is expressed as a percentage relative to that in empty plasmid-containing cells. Results are graphed as the means ± s.d. for triplicate samples. The data presented are representative of three independent experiments.

S6 Fig

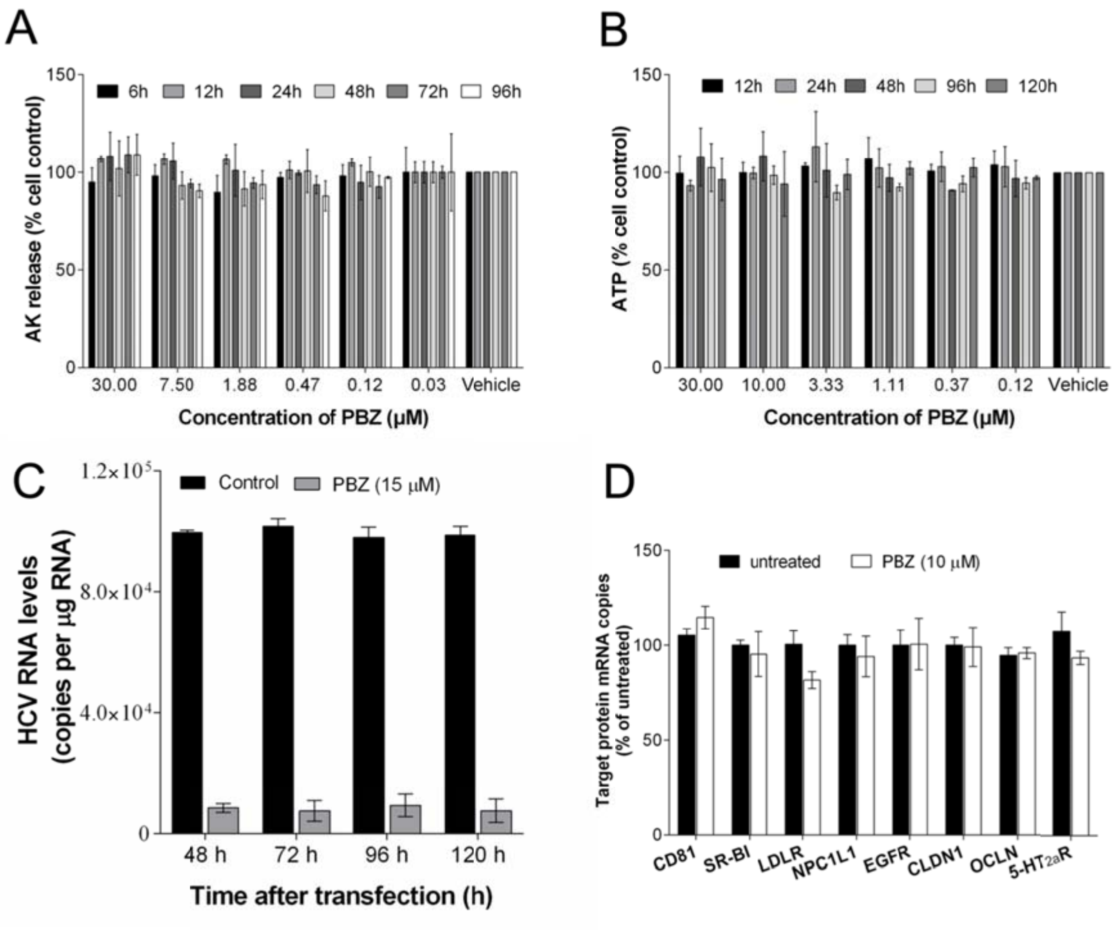

S6 Fig. PBZ is non-cytotoxic, does not affect cell proliferation and does not affect the transcription of known HCV receptors on Huh7.5.1 cells. (A, B) At indicated time post-treatment, culture supernatant was harvested and PBZ induced toxicity was determined by measuring cellular release of adenlyate kinase (AK) using the The ToxiLight™ bioassay kit **(A)**, or cultures were lysed and bioluminescence detection of cellular ATP was performed using the The ATP Determination Kit as a quantitative measure of cell viability **(B)**. Results are shown as a percentage of relative luminescence (RLU) compared to vehicle-treated cells. **(C)** PBZ inhibit HCV proliferation. Huh7.5.1 cells were treated with 15 μM PBZ followed by HCVcc infection

over the course of 5 days. All results are graphed as the means ± s.d. for triplicate samples. **(D)** PBZ treatment does not negative the transcription of other reported HCV receptors/entry factors. Huh7.5.1 cells were treated with PBZ (10 μM) for 48h. The mRNA copies of CD81, SR-BI, LDLR, NPC1L1, CLDN1, EGFR and OCLN were evaluated by qRT-PCR. The data are expressed as a percentage relative to 0.5% DMSO-treated cells as controls. All results are graphed as the means ± s.d. for triplicate samples.

S7 Fig

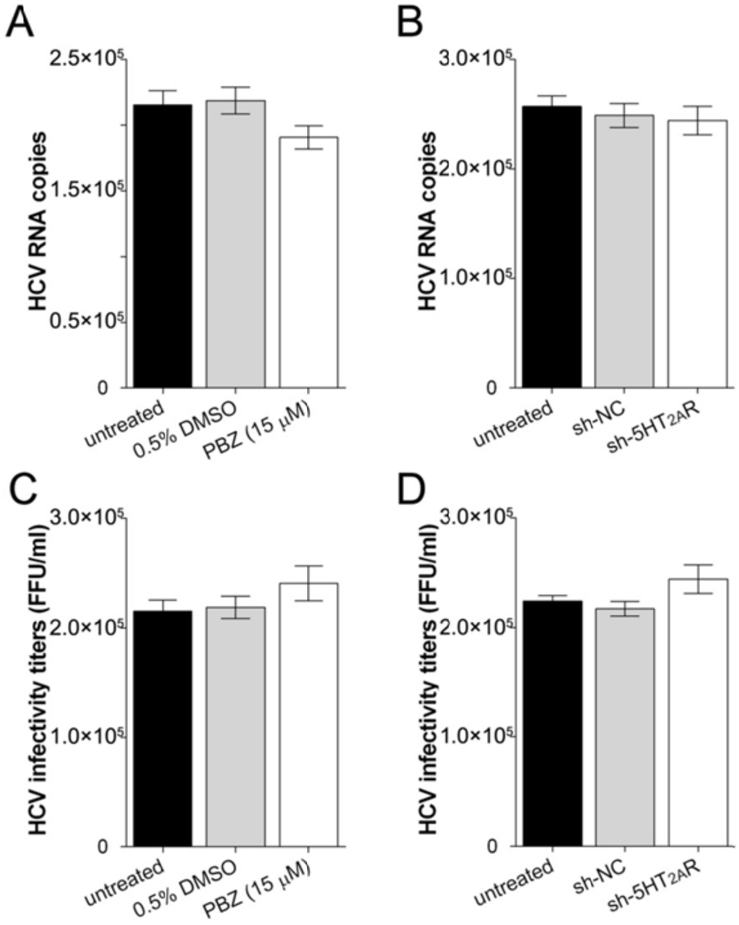

S7 Fig. PBZ treatment and the silencing of 5-HT$_{2A}$R do not inhibit full-length HCVcc RNA replication or secretion of infectious HCVcc. Huh7.5.1 cells chronically infected with HCVcc were treated with 0.5% DMSO or 15 µM PBZ, or were reverse transfected with 0.8 µg of sh-NC or sh-5HT$_{2A}$R. **(A, B)** At 48 h PBZ treatment or post-transfection, total intracellular RNA was extracted. HCV RNA levels were quantified by qRT-PCR and normalized to host GAPDH. **(C, D)** At 48 h PBZ treatment or post-transfection, HCVcc infectivity titers were determined. Results are graphed as the means ± s.d. for triplicate samples.

S8 Fig

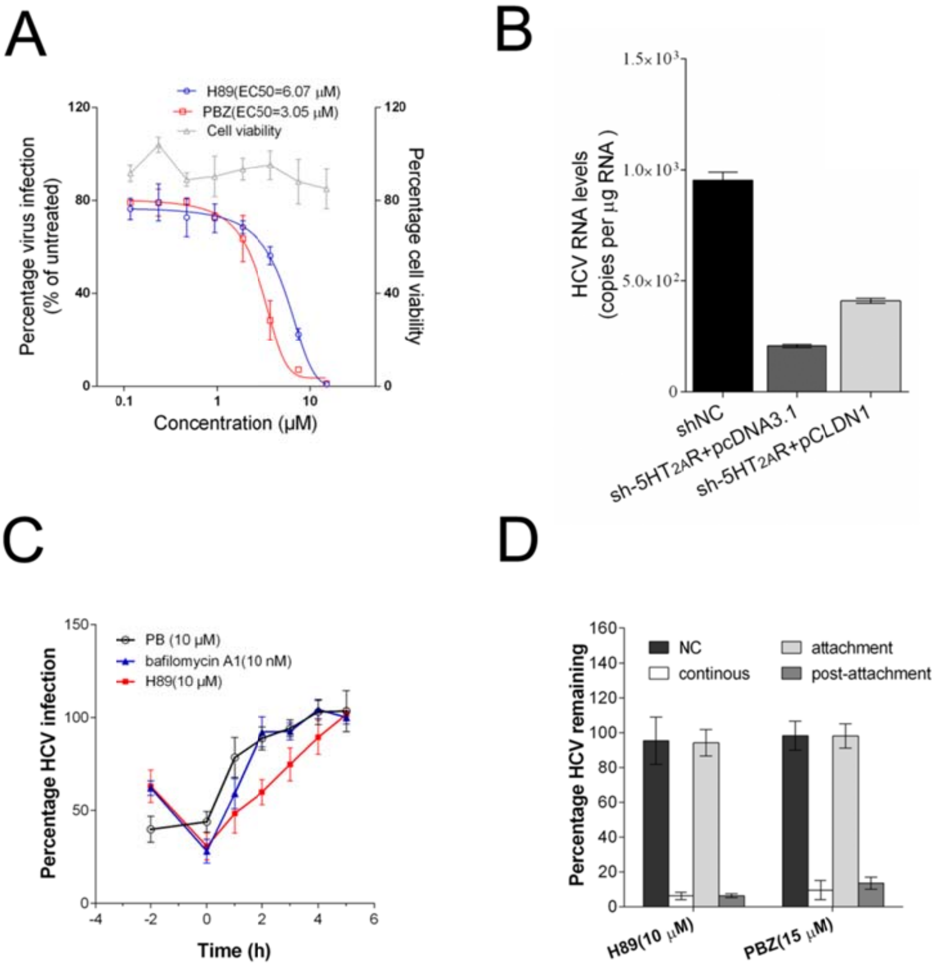

S8 Fig. H89 plays the same functions in HCV proliferation like PBZ. (A) Inhibitory activities of PBZ (red line) and H89 (blue line) on HCVcc. Cells infected by HCVcc were treated with PBZ or H89 at the indicated concentrations at 37 °C for 48 h. Virus infection and cell viability are expressed as percentages relative to 0.5% DMSO-treated control cells. **(B)** HCVcc infection is rescued by CLDN-1 overexpression. Huh7.5.1 cells containing sh-NC, sh-5HT$_{2A}$R and pcDNA empty plasmid, or sh-5HT$_{2A}$R and pCLDN-1 were infected by HCVcc at 37 °C for 48 h. Virus infection are shown HCV RNA levels, determined by RT-qPCR and normalized to GAPDH, at the triplicate times after infection. **(C)** The kinetics of HCV inhibition mediated by

PBZ or H89 was determined by time-of-addition assays. Huh7.5.1 cells were incubated with HCVcc at 4 °C for 2 h (T = -2). At different time points (T = -2 to T = 5), PBZ (10 μM) and H89 (10 μM) were individually added to the cells at 37 °C for 2 h. **(D)** H89 inhibits the post-attachment events. Huh7.5.1 cells were infected with HCVcc and incubated at 4 °C for 2 hours. Unbound virus was removed by two washes with cold media. Fresh medium was subsequently added, and the cells were shifted to 37 °C to allow synchronous infection. PBZ (10 μM) and H89 (10 μM) were provided in the media either continuously, during the 4 °C incubation only (initial attachment), or during the 37 °C incubation phase only (post-attachment). Virus infection is expressed as a percentage relative to control cells. All results are graphed as the means ± s.d. for triplicate samples.
